# Introduction to Single-Cell DNA Methylation Profiling Methods

**DOI:** 10.3390/biom11071013

**Published:** 2021-07-10

**Authors:** Jongseong Ahn, Sunghoon Heo, Jihyun Lee, Duhee Bang

**Affiliations:** 1 Department of Chemistry, Yonsei University, Seoul 03722, Korea; jongsungahn33@gmail.com (J.A.); 2011133049y@gmail.com (S.H.); 2 Department of Clinical Pharmacology and Therapeutics, College of Medicine, Kyung Hee University, Seoul 02447, Korea; 3 Department of Biomedical Science and Technology, Kyung Hee University, Seoul 02447, Korea

**Keywords:** DNA methylation, single cell, bioinformatics

## Abstract

DNA methylation is an epigenetic mechanism that is related to mammalian cellular differentiation, gene expression regulation, and disease. In several studies, DNA methylation has been identified as an effective marker to identify differences between cells. In this review, we introduce single-cell DNA-methylation profiling methods, including experimental strategies and approaches to computational data analysis. Furthermore, the blind spots of the basic analysis and recent alternatives are briefly described. In addition, we introduce well-known applications and discuss future development.

## 1. Introduction

In humans, DNA methylation is only found on cytosine residues [1]. DNA methyltransferases (DNMTs) transfer the methyl group of S-adenyl methionine (SAM) to the fifth carbon of the cytosine residue to form 5-methylcytosine [2], commonly referred to as 5mC or mC. Methylation of vertebrate genomes occurs on CG dinucleotides (commonly referred to as CpG to emphasize the importance of order) [1]. The methylation of non-CpG sites is only observed in the brain and in stem cells [3,4]. Therefore, detecting DNA methylation is often the same as confirming whether the cytosine of CpG in the genome is methylated. In humans, 28 million CpG dinucleotides are present in the genome, and 60–80% of those sites are known to be methylated [5,6]. This is certainly a large number, but compared to other dinucleotides, CpG is underrepresented [7]. As a result, the distribution of CpG in the genome is sparse at most locations but dense at some locations. Among the regions where CpGs are distributed at high density, the regions that satisfy the CpG density and scale criteria are called CpG islands (CGIs) [8,9]. Most housekeeping genes are present near CGIs [10], and there is a high correlation between the location of CGIs and promoters [11]. Studies have shown that CGIs function as genomic platforms for regulating transcription at their associated promoters [12]. Therefore, some profiling methods enrich high CpG regions such as CGIs [13]. 

In general, methylation of the CpG site is often taken as a sign of repression of gene transcription [14]. The relationship between DNA methylation and repression of gene expression has been reported in several studies and occurs in genomic imprinting [15], X-chromosome inactivation [16], and silencing of retroviral elements [17]. Nevertheless, not all methylation implies repression. For example, it has been observed that methylation of the gene body is associated with the expressed gene [18]. Other exceptions have also been reported [19]. These counterexamples suggest that DNA methylation can reveal important implications not only for CGIs, but also for numerous other sites. Therefore, methods such as reduced representation bisulfite sequencing (RRBS) [20], which focus mainly on high-CG regions, have the risk of missing important features. Therefore, a method such as whole-genome bisulfite sequencing (WGBS) [3] might be useful for observing the entire genome. On the other hand, counterexamples also suggest that CpG methylation of a specific site or region does not guarantee gene repression. The precise meaning of CpG methylation for a gene can be confirmed when cross-validated with other information, such as RNA expression levels or chromatin accessibility. For this reason, many multi-omics methods have been developed. However, this does not mean that profiling only methylation is meaningless. DNA methylation has the advantage of showing easily observable differences between samples under different conditions. By comparing the relative methylation between samples, it is possible to determine the key specific CpG sites or regions responsible for the differences. The terms hypermethylation and hypomethylation are used to refer to the relative increase or decrease in methylation, respectively, in such comparisons [21]. Although the methylation status of most CpG sites remains unchanged, a single CpG site or multiple CpG sites in a genomic region may become hypermethylated or hypomethylated as the environmental conditions change [22]. Under each condition, it has been reported that hypermethylation or hypomethylation occurs at different CpG sites [23,24,25,26,27,28,29]. Individual single cells also show differences in methylation at CpG sites [30], which is due to cell differentiation [31,32]. At the single-cell level, the methylation of individual CpG sites is clearly divided into methylated and unmethylated, and methylation of different locations is simply showing differences between different cells. Therefore, in the single-cell methodology, DNA methylation can be an efficient marker, distinguishing individual cells under different conditions or different cell types.

In this review, we describe methods for DNA methylation profiling in single cells. We introduce the basic principles and technologies shared between single-cell profiling methods and discuss the differences between methods. Although there are some profiling methods for modifications of 5mC, such as 5-hydroxymethylcytosine (5hmC), 5-formylcytosine (5fC), and 5-carboxylcytosine (5caC), these are not covered here. In addition, we only introduce genome-wide methods, as most single-cell methylation analyses require data from many locations within the genome for comparison. Because the main purpose of methylation analysis is to search for differences between groups, mainstream data analysis of single-cell methylation data does not greatly differ from bulk methylation data analysis. Therefore, we introduce a standardized basic methylation analysis process. Although many multi-omics methods are introduced, the individual analytical processes of each are beyond the scope of this review and, therefore, not discussed; this includes analyses such as RNA and chromatin accessibility. Instead, we describe the problems that researchers often encounter in the process of basic analysis and recent efforts to solve them. Additionally, we introduce an application method where DNA methylation is mainly used in single cells. Finally, we discuss the future direction of development.

## 2. Experimental Methodologies

### 2.1. Bisulfite Conversion

**Figure 1:**
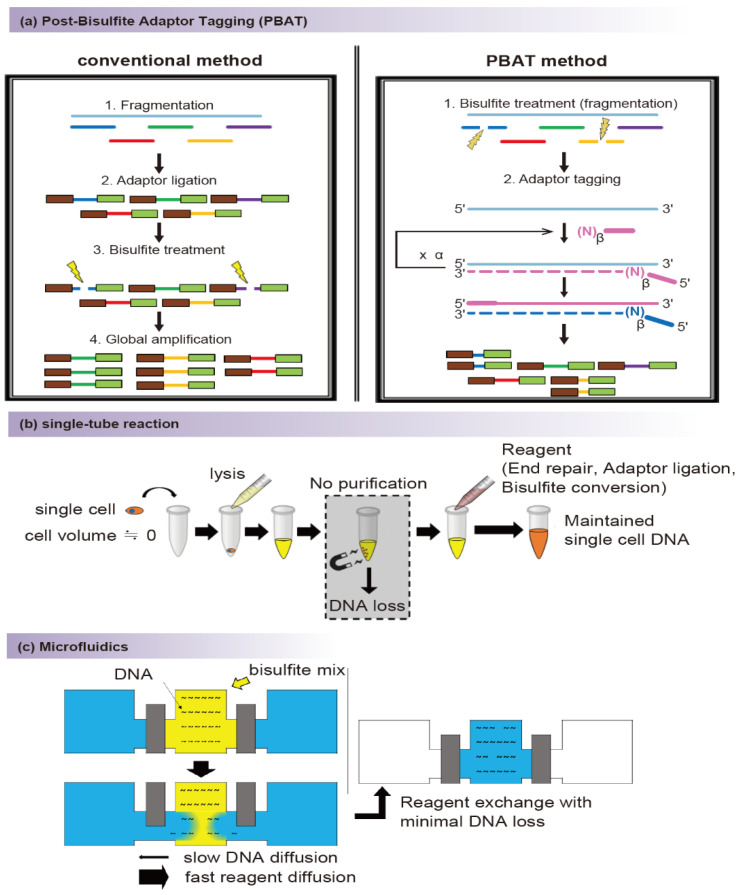
Key strategies to minimize DNA loss in single-cell DNA methylation profiling methods. Because a single cell contains a small amount of DNA, several methods are used to minimize loss. (**a**) Overview of the post-bisulfite adaptor tagging (PBAT) method to prevent loss due to degradation during the bisulfite conversion process. Unlike the conventional method (left panel), loss of shortened DNA fragments is prevented in PBAT (right panel). Each single-cell methylation profiling method using the PBAT strategy differs in the number of amplifications of the bisulfite conversion product (α) and the number of random sequences in the primer (β). (**b**) Overview of single-tube reaction. Common to several methods, reagents are continuously added, without purification, to the tube or well containing cell lysate or nuclei. In this way, DNA loss during the purification process can be prevented. (**c**) Use of microfluidics demonstrated in the microfluidic diffusion (MID)-based reduced representation bisulfite sequencing (RRBS) process. DNA loss can be minimized during purification using microfluidics. This figure is based on the figure of a previous article (Ma, S., de la Fuente Revenga, M., Sun, Z. et al. 2018) [41] and adapted with permission from 2018 Springer Nature.

There are several methods of profiling methylation, and these can be roughly divided into two groups depending on whether or not base conversion occurs. In the case of base conversion, cytosine or methylcytosine is selectively modified by reagents and finally detected as thymine by sequencing. The base-conversion–based method has the advantage of allowing inspection of methylation at individual CpG sites, whether converted or not. Methods of this type include bisulfite sequencing [33], ten-eleven translocation (TET)-assisted pyridine borane sequencing (TAPS) [34], and enzymatic methyl-seq (EM-seq) [35]. Because TAPS and EM-seq were recently developed, the conventional single-cell methylation profiling was performed based on bisulfite conversion. The method using bisulfite conversion is considered the gold standard for profiling DNA methylation [36]. It was favored by researchers because of its high conversion efficiency (>99%) [37], reproducibility [38], and simple accessibility through a commercial kit [39]. However, the bisulfite conversion method employs harsh reaction conditions that cause DNA degradation. Degradation causes loss of DNA and reduced sequence quality, which results in loss of final data yield [37]. Post-bisulfite adaptor tagging (PBAT) [40] was developed to solve the problem of loss caused by degradation (Figure 1a).

Although PBAT cannot prevent degradation by bisulfite conversion, it prevents loss in the next-generation sequencing (NGS) library preparation process by ligation of the adaptor after degradation. Therefore, it is widely used in the single-cell methylation profiling method, where loss must be minimized due to a small amount of input.

#### 2.1.1. RRBS-Based Methods

**Figure 2:**
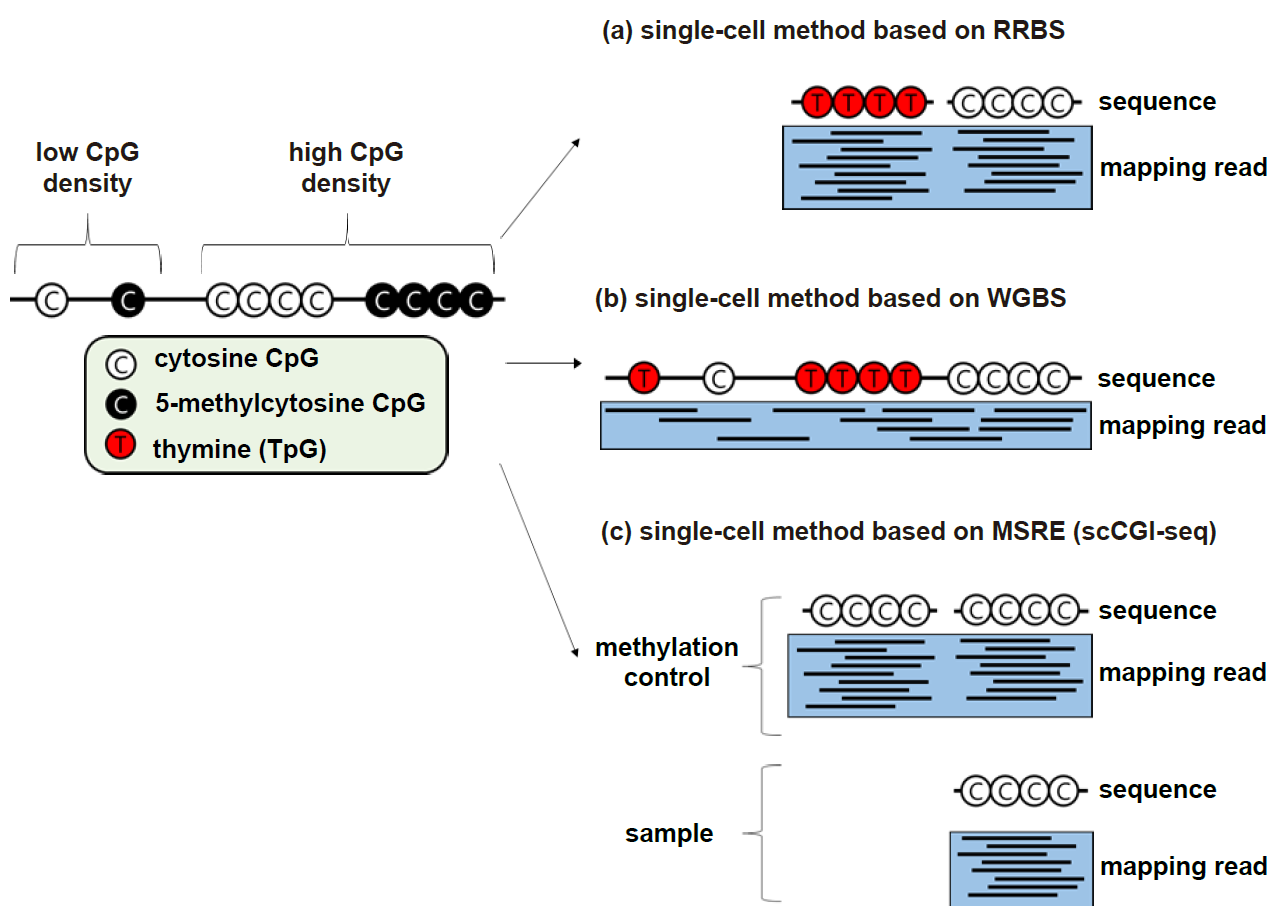
The pattern of the resulting data that can be obtained for each basic method. The resulting pattern of each single-cell profiling method reflects the pattern of the basic method associated with it. The figure format was based on the figure in the paper (Ja-Rang Lee, et al. 2018) [45]. (**a**) An example of the final data pattern represented by the single-cell method based on RRBS. According to the basic principle of RRBS, reads are observed mainly at high-CG positions. (**b**) An example of the final data pattern represented by the single-cell method based on WGBS. According to the basic principle of WGBS, a relatively even distribution of reads is observed. (**c**) Example of the pattern of results in single-cell (sc)CGI-seq where methylation-sensitive restriction enzymes (MSREs) are used. Similar to RRBS, the read is observed at the high-CG position in the genome, but the methylated site can be observed based on the difference between the control and the sample.

RRBS [20] and WGBS [42,43] are popular genome-wide methylation profiling methods. Both methods include bisulfite conversion and NGS preparation. The main difference between the two methods is that RRBS screens GC-rich regions using appropriate restriction enzymes and size selection (Figure 2a) [44].

Because most CpG sites do not show significant changes in methylation status, RRBS can be useful for cost savings [22]. Single-cell (sc)RRBS [46], quantitative (Q)-RRBS [47], and microfluidic diffusion (MID)-RRBS [41] are methods that optimize RRBS for single-cell research (Table 1).

Traditional RRBS can be broadly divided into five steps. The first is to digest DNA using restriction enzymes, the second is adaptor ligation and its preprocessing, the third is GC-rich site enrichment using gel size selection, the fourth is bisulfite conversion, and the last is the amplification process before sequencing. Several purification steps are included in these processes, and the final step, the amplification process, exists to compensate for losses during the process. Unlike previous RRBS, RRBS in a single cell is not free from loss. Due to the small amount of input DNA from a single cell, the conventional method does not guarantee that the target DNA is maintained until the final amplification process. Therefore, single-cell RRBS has been refined in the direction of reducing loss. scRRBS is a method that focuses on minimizing the losses that occur during the purification process. Loss reduction is achieved by restriction enzyme digestion, adaptor ligation processes, and bisulfite conversion in a single tube without purification (Figure 1b). In addition, the use of tRNA carriers and reordering of size selection are also aimed at preventing loss. The quantitative (Q)-RRBS method introduced unique molecular identifiers (UMI) to overcome the bias problem of multiple cycles of PCR in order to amplify the small amount of DNA in scRRBS. If the length of the UMI is sufficient, reads with the same UMI sequence can be regarded as duplicated reads derived from the same molecule. The Q-RRBS method may be a good choice for researchers who are concerned about duplications and artifacts. MID-RRBS is identical to scRRBS in that the goal is to reduce DNA loss. However, there are differences in the process identified as the cause of the loss and the prevention method. The MID-RRBS method pointed to the column purification process during bisulfite conversion as the main cause of loss and suggested an alternative using microfluidics technology (Figure 1c). Using the microfluidic device can replace the reagents and DNA purification at the same time. The slow diffusion rate of DNA according to molecular weight enables the minimization of DNA loss. Single-cell triple-omics sequencing (scTrio-seq) [48] and single-cell methylome and transcriptome sequencing (scMT-seq) [49] are multi-omics methods based on scRRBS. In both methods, the nucleus is maintained through a mild lysis protocol to prevent DNA and RNA from mixing and is then physically separated. The separated DNA is subjected to scRRBS, and in the case of RNA, scTrio-seq is performed by scRNA-seq [62], and scMT-seq is performed using the Smart2-seq [63] protocol. Both methods were able to confirm the correlation between methylome and transcriptome, but scTrio-seq showed that copy number variations (CNVs) can also be estimated using DNA fragments.

#### 2.1.2. WGBS-Based Methods

Although RRBS is sufficient in many cases [64], there are several limitations due to the nature of screening in only a few genomic regions [13,65]. On the other hand, WGBS (specifically methylC-seq [43]) has the advantage of being able to cover most of the CpGs in the genome (Figure 2b). Compared to RRBS, which has to undergo several purification and selection processes, WGBS undergoes a relatively simpler process. Therefore, it is considered relatively important in WGBS to prevent loss by degradation in bisulfite conversion, and thus many WGBS-based single-cell methods are often modified based on PBAT [40]. The PBAT method is divided into four steps. The first process is bisulfite conversion, and the second process synthesizes the first DNA strand from the converted DNA with a primer containing biotin at one end and a random tetramer (N_4_) at the other end. The next step is to immobilize the synthesized DNA with streptavidin and then synthesize the second DNA strand using a primer containing a random tetramer. Finally, a second DNA strand of the appropriate size is selected and sequenced.

WGBS-based single cell profiling methods include single-cell bisulfite sequencing (scBS-seq) [50], single-cell PBAT (scPBAT) [51], single-cell WGBS (scWGBS) [52], single-nucleus methylcytosine sequencing (snmC-seq) 2 [53], and single-cell combinatorial indexing for methylation analysis (sci-MET) [54]. These methods also generally follow the flow of PBAT, but there are differences in the details.

The scPBAT method is almost identical to the original PBAT, except that it uses a primer containing a random tetramer (N_4_) and a weak (A, T, or U) tetramer (W_4_). However, since the original PBAT method does not have an amplification process, there is a limit to its applicability to a small amount of single-cell DNA. As a result, scPBATs can only be used for limited applications, such as determining the methylation of repetitive elements. Therefore, the most recent version of the scBS-seq method is based on PBAT, but unlike PBAT, the bisulfite conversion in scBS-seq is done directly in the cell lysate. The primer also contains a random hexamer (N_6_) instead of a random tetramer. PBAT only performs annealing and extension in the two-strand formation steps. However, scBS-seq performs five cycles of amplification on bisulfite-converted DNA in the first DNA strand synthesis step. The second strand synthesis is performed only once, as in the original PBAT, with the difference that 10~15 cycles of amplification are performed using adaptor sequences present on both sides of the synthesized DNA. In these procedures, the purification process using streptavidin beads is omitted and replaced with solid-phase reversible immobilization (SPRI) beads. scWGBS uses a primer containing a random hexamer for the synthesis of the first DNA strand, similar to scBS-seq, but does not perform multiple cycles. Instead, terminal tagging occurs during the synthesis of the first strand, and the second strand synthesis is performed for up to 18 cycles using the tagged sequence. snmC-seq [66] and the improved snmC-seq2 [53] showed that the reaction proceeded in the nuclei instead of the previously used cell lysate. In addition, it showed relatively high coverage through the introduction of a single-strand NGS preparation method using an adaptase. It is similar to scWGBS in that the tagged sequence is used for second-strand synthesis. sci-MET tried to improve the scale by improving not only the use of nuclei, but also tagmentation through transposase and the performance of combinatorial indexing in multiple wells. In scWGBS and snmC-seq2, a tag sequence is added at the time of first-strand synthesis, but in sci-MET, the tag is inserted in advance during tagmentation by transposase.

Multi-omics methods are distinguished according to which methylation profiling method is combined with which other profiling method (RNA, chromatin accessibility). Single-cell genome-wide methylome and transcriptome sequencing (scM&T-seq) [56] and scTrio-seq2 [55], a variation of scTrio-seq [48], are based on scBS-seq. scM&T-seq is a combination of genome and transcriptome sequencing (G&T-seq) [67] and scBS-seq, and G&T-seq is a method of identifying both DNA and RNA based on Smart-seq2 [63]. RNA and DNA were physically separated through the G&T-seq process, and then the purified DNA was subjected to the scBS-seq process. scTrio-seq2 extended the observable range using scBS-seq instead of scRRBS in scTrio-seq. Therefore, like scTrio-seq, CNV estimation is possible.

On the other hand, techniques applied to the single-cell methylation profiling method, such as PBAT, can also be similarly applied to another method called nucleosome occupancy and methylome-sequencing (NOMe-seq) [68]. NOMe-seq can confirm open chromatin and CpG methylation in bisulfite-converted DNA using the difference in chromatin accessibility of GpC methyltransferase according to the presence or absence of nucleosomes. Single-cell chromatin overall omic-scale landscape sequencing (scCOOL-seq) [69], improved scCOOL-seq (iscCOOL-seq) [58], and scNOMe-seq [59] can monitor chromatin accessibility and CpG methylation together. If NOMe-seq is added based on scM&T-seq, it becomes single-cell nucleosome, methylation, and transcription sequencing (scNMT-seq) [57]. This method makes it possible to confirm chromatin accessibility, DNA methylation, and transcriptome profiling.

### 2.2. Conversion-Free Methods

Observation of methylation by methods other than conversion is largely divided into two classes: the use of affinity binding of methylcytosine and the use of the sensitivity of the restriction enzyme to methylcytosine. Methyl-CpG-binding domain sequencing (MBD-seq) and methylated DNA immunoprecipitation sequencing (MeDIP-seq) are representative affinity-based methods [70,71]. Affinity-based methods are not suitable for application on a single-cell scale, because these methods generate average DNA methylation profiles based on DNA fragments, which does not allow discrimination of differences in DNA methylation patterns across single cells [72]. Therefore, until now, application in single cells has not been within the range of our knowledge. MRE-seq [73] is a representative example of a method using the sensitivity of a restriction enzyme to methylcytosine. The unmethylated CGIs are sequenced after a size-selection process. Similar to affinity-based methods, MRE-seq was not suitable for single cells because it required the enrichment of DNA fragments. However, the MSRE-based method could be improved, unlike the affinity-based method. Since the affinity-based method determines the presence or absence of methylation by enrichment of DNA fragments with methylcytosine, it is difficult to improve it in a single cell with a small amount of DNA. On the other hand, since the MSRE-based method determines whether methylation occurs by cleavage, the enrichment of the DNA fragment is an intermediate step for detection. A refinement of the single-cell method using MSRE can be found in Methyl-seq [74]. Methyl-seq is similar to MRE-seq, but it compares the results with methylation-sensitive and -insensitive restriction enzymes. This method was not developed for single-cell purposes. scCGI-seq [60] measures methylation in a similar way to methyl-seq. If the CGI site is methylated, cleavage by MSREs is inhibited, the sequence is amplified and detected, and the result is compared to the result of digestion by a methylation-insensitive restriction enzyme (Figure 2c). Multiple displacement amplification (MDA) makes it possible to use this approach on a single-cell scale.

Although the method using MSRE continues to develop [75], there is no conversion, so the GC rate is maintained and there is no degradation. However, despite these advantages, it is clear that the bisulfite conversion method is the standard at this time. Therefore, the next chapter describes a standardized analysis method, assuming the use of bisulfite conversion.

## 3. Data Analysis

### 3.1. Data Quality Assessment

After sequencing experiments, including RRBS or WGBS, preprocessing of sequence data is required. Preprocessing steps can be divided into data quality control (QC), trimming of sequencing reads, and alignment of sequencing reads. The first part of quality control measures overall basic sequencing data quality utilizing software programs such as FastQC [76]. After overall sequence quality assessment, the conversion efficiency of bisulfite sequencing should be confirmed. For samples with low bisulfite conversion efficiency, distinguishing bases sequenced as cytosines in CpG sites from true methylated cytosines or sequencing error is challenging. This acts as a confounding factor in downstream analysis, including finding differentially methylated regions. The conversion efficiency can be directly derived from unmethylated lambda DNA spiked in by calculating the fraction of converted bases at cytosine sites or using available software programs [37,77,78]. 

### 3.2. Read Trimming and Sequence Alignment

Artifactual sequences, including sequencing adaptors and homopolymeric sequences in sequence read ends, are trimmed using software such as Trim Galore! [79], fastp [80], and Trimmomatic [81]. The trimmed sequence reads are then aligned on the reference genome. The cytosine-to-thymine conversion causes two problems in sequence alignment. The first is the alignment of converted thymines onto original cytosine residues. The purpose of the alignment step is to identify the most likely genomic region from which a given sequence read originated. To discover such regions, alignment software adopts a penalty score that is correlated with the number of mismatches. The converted cytosines are considered mismatches in the alignment step. Therefore, the alignment should consider these high penalty scores. The second problem is that the alignment must be unique and accurate, although the complexity of a sequence read is low. Bisulfite sequence reads are AT-rich. This causes increased similarity between reads although the sequences originated from different genomic regions. This hinders the unique alignment of each read. There are many aligners designed to solve these problems (Table 2).

**Figure 3:**
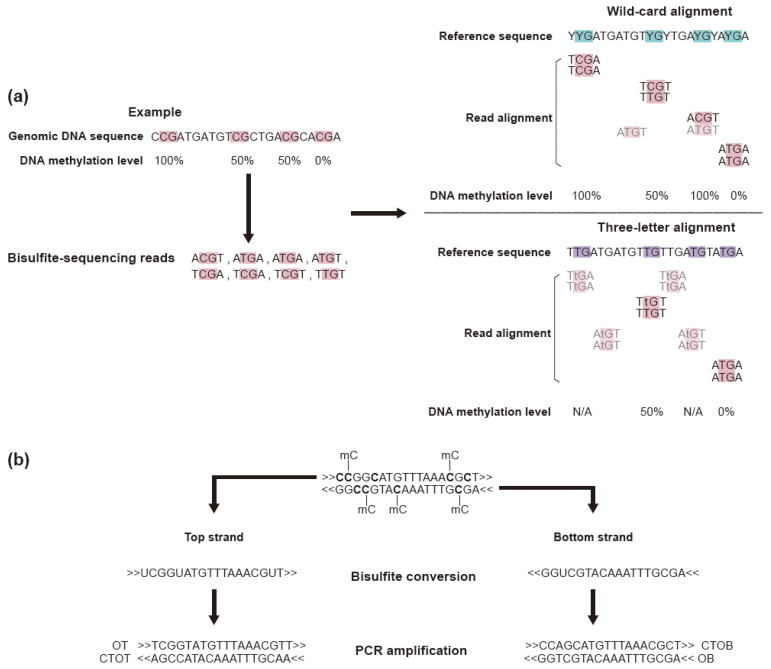
The bisulfite treatment-based sequencing data analysis should take into consideration the converted normal cytosine. (**a**) With the exception of the CpG loci (red), all cytosine residues of produced sequence reads are converted to thymine after PCR (left panel). These sequence reads are aligned on the reference genome using the wild-card method (upper right panel) or three-letter method (lower right panel). Although the wild-card alignment aligned more reads and coverage is increased, the methylation level is biased. In the three-letter system, some reads failed to align but the calculated methylation level is unbiased compared to the wild-card method when there is alignment (transparent reads are an alignment failure) (adapted with permission from [82], 2012 Springer Nature). (**b**) Sequence reads after bisulfite treatment. Due to the PCR step, four types of sequence reads are produced. Two are from the original target molecule (OT, OB), and the other two are from the complementary strand generated by PCR (CTOT, CTOB). OT: original top, OB: original bottom, CTOT: complementary to original top, CTOB: complementary to original bottom (adapted with permission from [83], 2012 Springer Nature).

Aligners can be classified as either utilizing a wild-card character for alignment or adopting a three-letter system for reference genome indexing [82]. The former converts all cytosines with wild-card character Y (cytosines and thymines), and the latter utilizes a reference genome in which all cytosines are converted to thymines in both strands. Although the wild-card method has the benefit of increased coverage resulting from more surviving reads compared to the three-letter system, the biased estimation of methylation rate due to misalignment is a major issue. On the other hand, the three-letter system has reduced coverage because it discards more reads than the wild-card system. However, the bias in methylation rate is lower than for the wild-card system (Figure 3a). 

Misalignment in both methods arises from repeat sequences, such as transposable element sequences. Because the accuracy of alignment in these regions can be further improved using long-read sequencing technologies, the recent design of alignment software focuses on low-computation resources and low time complexity of alignment [84,85,86]. For the downstream analysis after alignment, such as single-nucleotide polymorphism (SNP) discovery in CpG sites, researchers should consider if the bisulfite sequencing data are a mixture of reads originating from the original top and bottom strands of DNA molecules and their complementary sequences generated by PCR (Figure 3b).

### 3.3. Methylation Analysis Using Methylation Level

The major objective of methylation analysis is to explore epigenetic evidence that constitutes differences between samples, organs, and disease status, including cancer [87,88,89]. For the purpose of discovering these differences, a numeric value that implies this concept is required.

(1) $$\beta =\;\frac{Methylated\;Cytosine}{Methylated\;Cytosine+Unmethylated\;Cytosine}$$

(2) $$M=\log_{2}\left(\frac{\max\left(Methyl,\;0\right)+\;\alpha }{\max\left(Unmethyl,\;0\right)+\;\alpha }\right)\;$$

**Figure 4:**
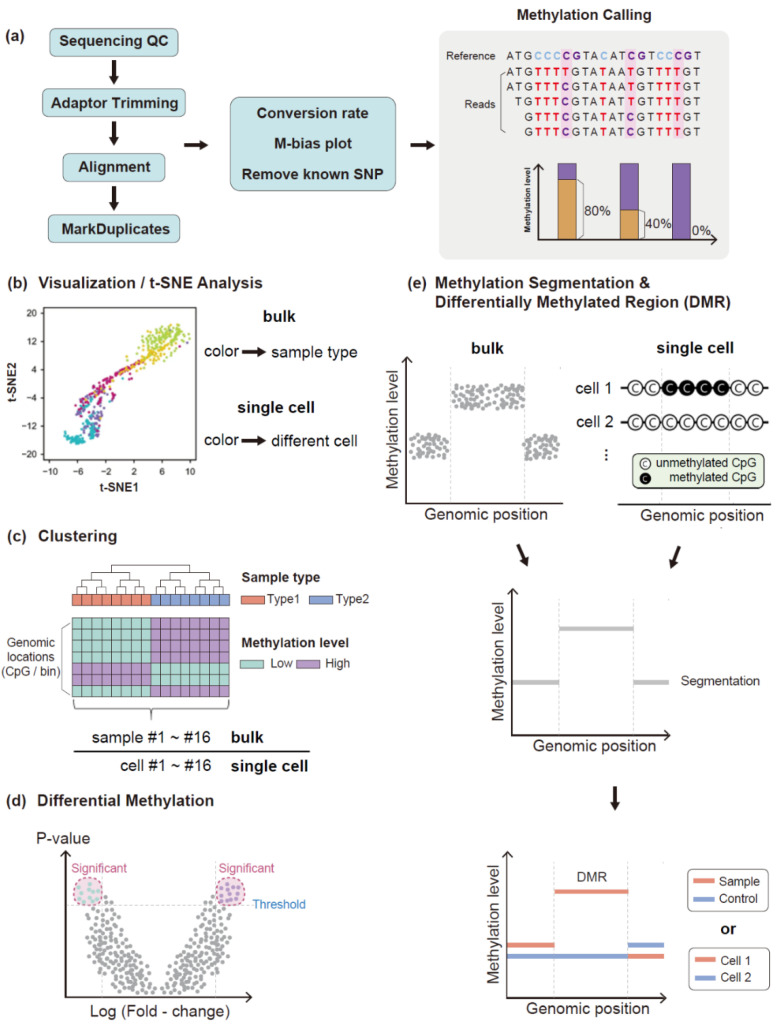
Analysis methods of DNA methylation. (**a**) Overview of methylation analysis pipeline. The analysis starts with the quality check of raw sequence reads followed by adaptor trimming and alignment. After alignment, two tracks of analysis are performed. The first is assessment of the experimental quality, such as bisulfite conversion rate, M-bias plot, and removal of known single-nucleotide polymorphisms (SNPs). The second is removal of duplication followed by methylation calling. After methylation calling, several steps, such as visualization (**b**), cluster analysis (**c**), and identification of differentially methylated sites or genes between bulk or single-cell groups (**d**) or regions (**e**), are carried out. Each analysis method is used on both the bulk scale and single-cell scale, and individual single cells are treated similarly to individual samples in bulk. The image in (**b**) was adapted from the t-SNE figure of the open access iscCOOL-seq paper (Gu, C., Liu, S., Wu, Q. et al. 2019) [58]. t-SNE: t-stochastic neighbor embedding.

A widely used term is the β-value (Equation (1)), which ranges from 0 to 1 and is defined as the proportion of methylated cytosines at a given CpG site. Although this value is biologically interpretable, another metric, the M-value (Equation (2)), is used for statistical interpretation. This methylation-calling step is the initial step of methylation analysis (Figure 4a). Although the β-values and M-values range in bulk methylation level analysis, these values are usually binary (unmethylated or methylated) in single-cell analyses.

After methylation calling, subsequent analyses such as t-stochastic neighbor embedding (t-SNE) (Figure 4b) for visual analysis, cluster analysis (Figure 4c), and identification of differentially methylated cytosines (DMCs) (Figure 4d) or differentially methylated regions (DMRs) (Figure 4e) are conducted. Selection from DMCs and DMRs for certain analyses is dependent on the feature a researcher considers [90]. In the case of analysis focusing on DMRs, there are additional numerical values to distinguish differences between groups that summarize the methylation information in a region, including average methylation fraction (AMF) or individual methylation fraction (IMF). A recent study imported the concept of haplotype block [91]. This method builds a haplotype block of CpG sites, considering each CpG site as an SNP locus and the methylation status of each CpG site as B-allele frequency (BAF). To build haplotype blocks, the authors compiled WGBS data and RRBS data for various types of tissues. To explore differences between tissues, the authors suggested a new term, methylation haplotype load (MHL). This metric performed best compared to AMF and IMF for tissue classification and detection of tissue of origin for cell-free tumor DNA samples [91]. These various analysis methods are similarly used in single-cell analysis. As an example, two papers [92,93] analyzed methylation of circulating tumor cells (CTCs) and clustered single-cell groups and found DMR. Researchers can find the difference by applying the above methods even in single cells.

### 3.4. Methylation Analysis Using Methylation Pattern of Sequence Reads

**Figure 5:**
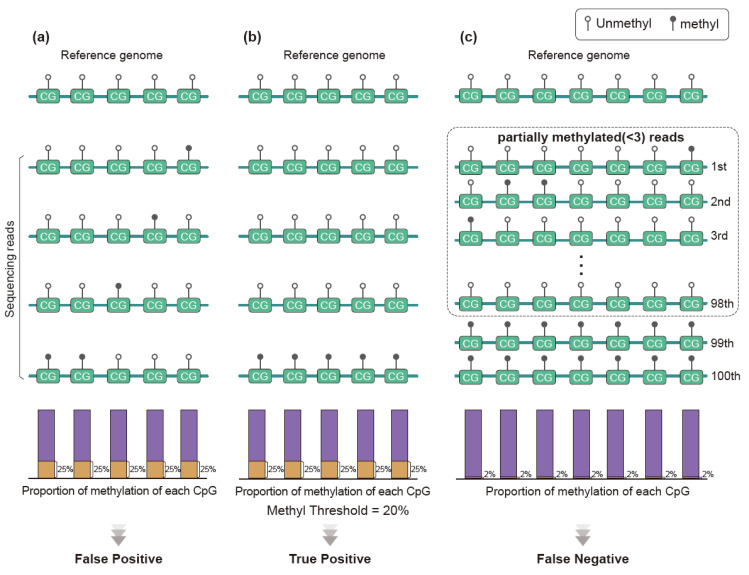
Benefits of read-pattern analysis. Single CpG loci values can confound the methylation call. Consider a scenario of detection of cancer using methylation. (**a**) Sequence reads in a given region are nearly identical to the reference pattern but are dissimilar due to an error of the methyltransferase. The overall methylation percentage is calculated as 25%. The methylation level of this region is different from that of the reference and, hence, a false positive methylation call can occur. (**b**) Although the CpG methylation level is the same in the former scenario, there is a molecule that is perfectly methylated. In this case, we consider that this region has a methylated molecule because the multiple CpG site error of methyltransferase in a single DNA molecule occurs with very low probability. Therefore, we call this true positive methylation. (**c**) In early cancer, there is a small cancer DNA burden in the blood that is nearly undetectable using single CpG methylation (here 2% of methylation). Unless utilizing the methylation pattern of molecules, a false negative result occurs.

Methods described above mainly rely on the methylation level of individual CpG sites. Recent methylation analysis makes use of the pattern of methylation in each read for disease diagnosis, especially for cancer [94,95]. This new concept in analysis is based on the biological property of methylation that there is a tendency to maintain methylation between adjacent CpG sites unless there is de novo methylation [96]. Conventional methods using β-values or AMFs are not suitable for disease detection when the disease burden is low. This is because those values “average out” the mixture of patterns that comprise the total information and low disease signals cannot be detected when the majority of signals are not disease signals (Figure 5).

**Figure 6:**
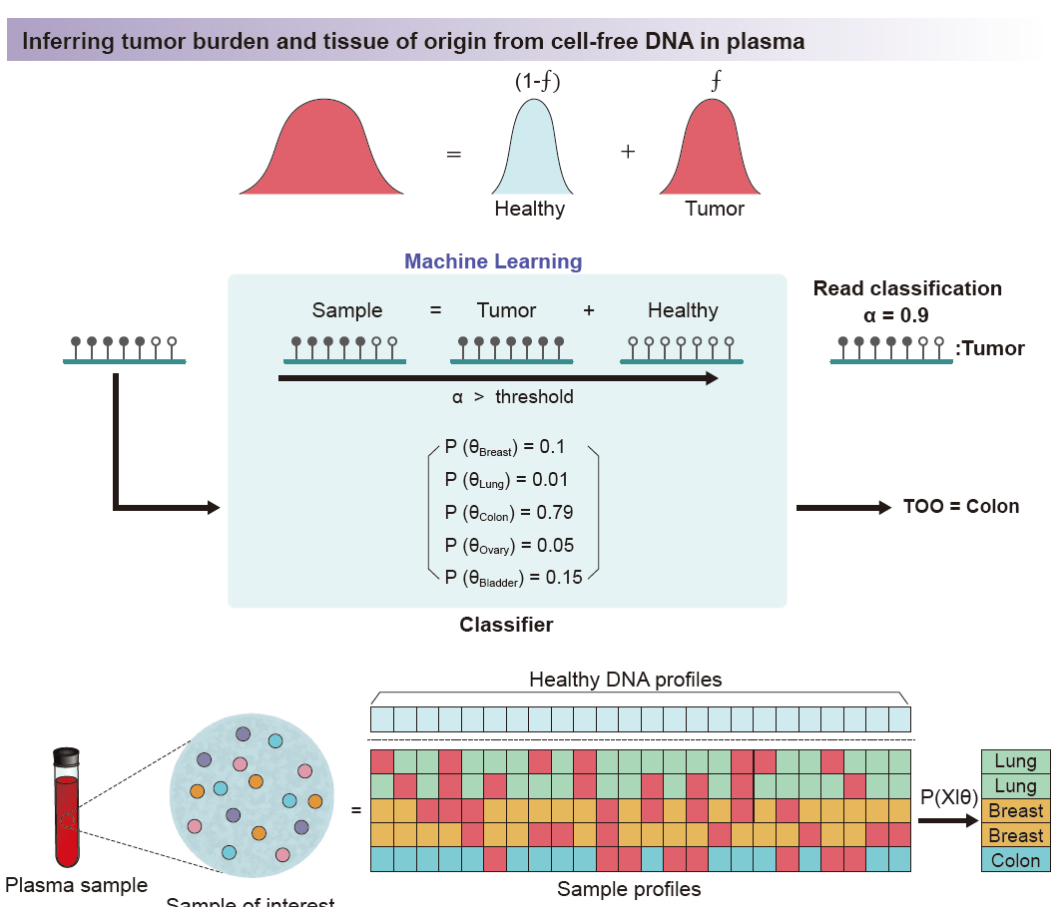
Applications of methylation pattern analysis in cancer clinical settings. Each DNA methylation pattern in a single sequence read is evaluated using a trained machine learning classifier. The classifier considers the methylation pattern as a mixture of a healthy (normal) methylation pattern and tumor methylation pattern. In the training step, both the normal methylation pattern and the methylation pattern information data for each cancer type are fed into the classifier. According to trained hyperparameters, the classifier scores each read and classifies whether the read (i.e., DNA molecule) originates from tumor or normal DNA. The model collects information for each read in an ensemble manner for each genomic region of interest. The collected signal is utilized to (1) decide whether the sample is cancerous and (2) deconvolute the tumor of origin. (TOO: tumor of origin, *f*: fraction of tumor).

The read-pattern method can detect DNA molecules having disease signals and has the possibility of increasing the chance of disease signal detection. Practically, liquid biopsy studies adopted this concept due to the very small amount of input materials extracted from samples. The concept of detection of DNA molecules “as is” by obtaining the pattern of methylation in each read becomes important in the early detection of cancer. A recent study using published clinical data mathematically shows that approximately 570 tumor DNA molecules are present in whole human blood from lung cancer with a primary tumor size of 1 cm^3^. Very limited amounts of blood samples (<15 mL) are accessible in clinical settings. This is equivalent to the detection of one or two DNA molecules [97]. Without the read-pattern analysis, methylation signals can be treated as negative (Figure 5c) in this setting. For example, a large liquid biopsy study has designed an ensemble classifier that categorizes the types of tumor based on the read-pattern analysis and showed remarkable results for the detection of early-stage cancer [94]. Moreover, the quantification of tumor-derived DNA molecules through the methylation pattern is an alternative method of observing tumor burden (Figure 6) [98,99]. 

These suggest that methods making decisions on the presence or absence of tumor DNA molecules by methylation read level pattern analysis are often intuitive compared to methods adapting average scores such as methylation rate. It has been reported that analysis using read-level methylation patterns in a single cell is possible because single-cell and liquid biopsies share a small amount of inputs [100,101]. Therefore, it can be considered as an option for those who wish to perform methylation analysis in a single cell.

## 4. Application

It is known that DNA methylation is an epigenetic marker that is inherited during cell division and affects the biological function of a cell [6,102]. In this review, we would like to briefly introduce the applications of single-cell DNA methylation studies, especially biological function studies, which use the fact that it is possible to find cellular heterogeneity, which is the advantage of single-cell sequencing over conventional bulk studies.

### 4.1. Cell Development

**Figure 7:**
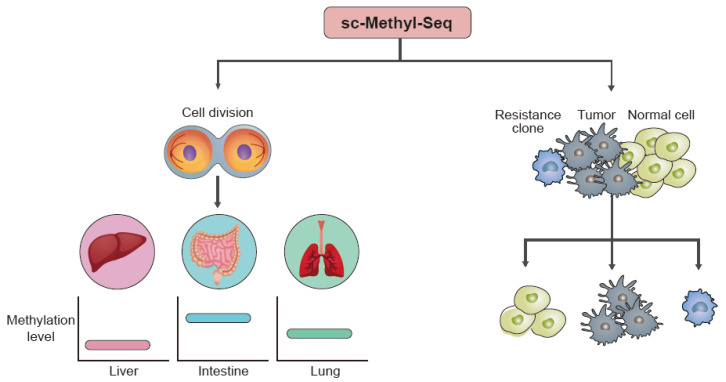
Application of single-cell methylation sequencing (sc-methyl-seq). Sc-methyl-seq can be applied to cell differentiation analysis (left panel) and rare-cell population analysis, such as a resistance clone in cancer (right panel).

The maturation of a germline cell or embryonic cell is affected by the expression of specific genes, which correlates with the methylation level in DNA. Ping et al. [103] found that tens of thousands of genomic loci are de novo methylated in preimplantation embryonic cells. This suggests that the balance between global demethylation and focal remethylation occurs in the preimplantation stage. At the same time, it was found that the paternal genome was demethylated faster and at a higher level than the maternal genome and that the paternal genome was more highly methylated than the maternal genome from the two-cell stage to post-implantation. Based on these methylation characteristics, they used single-cell methylation sequencing to investigate the mechanism of preimplantation cell methylation and its phenomena through a study of early blastomere lineage tracing. Preimplantation plays an important role in transforming terminally differentiated gametes into pluripotent cells through a mechanism that removes methylation during cell development. As a result of examining strand-specific changes using single-cell DNA methylation, it was observed that the loss of methylation maintenance was strand-specific [104]. CpG methylation was established broadly at the immature germinal vesicle stage using single-cell methylation sequencing to study not only the process of embryonic development (Figure 7, left) but also the process of human germ cell maturation. In particular, it was observed that non-CpG methylation continues to accumulate throughout the stage of maturation, suggesting that non-CpG methylation would have a different role than CpG methylation at the time of oocyte maturation [105].

### 4.2. Disease-Associated Studies

In patients with a disease, the pattern of DNA methylation has a different pattern than that of healthy individuals [106,107]. Among various diseases, cancer in particular has a DNA methylation pattern that a normal cell does not have that causes a difference in gene expression levels. In the case of studies on cancer with such heterogeneous properties, a multi-omics approach that analyzes genomic variation and RNA expression together is used rather than methylation alone. Researchers have developed a computational tool that learns present linkages between methylation and gene expression using gene- and cell-dependent features. With a multi-omics approach, the performance of certain analysis tasks such as clustering and cell type identification has improved [108]. A research group has recently developed a method called scTrio-seq2, which integrates single-cell RNA-seq and single-cell methylation sequencing data. For application, they have sampled multiple regions of colorectal cancer patients from the primary tumor to distal metastases to study and trace the lineage of cancer. They also co-profiled somatic copy number variations and showed the emergence of a sublineage. Through the integration of transcriptome data and methylation data, they have revealed the molecular association between gene expression and DNA methylation. Typically, the promoter regions were negatively correlated, but a positive correlation was observed in the gene body region. Each cancer sublineage showed a different DNA demethylation level across the whole genome [55]. Other research has analyzed the heterogeneity of liver cancer using single-cell methylation analysis. They found that the copy number change causes proportional changes in RNA expression but generally does not affect DNA methylation [48]. These examples show that the multi-omics method using single-cell methylation sequencing (sc-methyl-seq) can overcome the limitations of the previous method and have better discrimination capability (Figure 7, right). Thus, sc-methyl-seq can be used in various fields to address fundamental questions related to biological processes and diseases.

## 5. Future

Still, there are several problems with single-cell DNA methylation studies. The first among them is the degradation problem of bisulfite conversion, which is the current gold standard. However, at the single-cell scale where the amount is limited, the loss due to degradation is a more serious problem than at the bulk scale. To solve this problem, techniques such as PBAT are applied, yet the performance is not comparable to the methods using larger amounts of DNA [109]. In recent years, methods using TET enzyme activity, such as TAPS [34] and EM-seq [35], have been developed and are attracting attention as a solution to the chronic degradation problem. TET enzymes sequentially convert methylated cytosines into hydroxymethyl cytosines, formyl cytosines, and carboxyl cytosines [110]. The mild enzymatic reaction conditions of TAPS and EM-seq compared to bisulfite sequencing are free from DNA degradation issues. Prevention of degradation can be useful for other sequencing methods that read long-reads, such as single-molecule real-time (SMRT) sequencing or nanopore sequencing [111]. Therefore, TAPS or EM-seq may be considered an alternative to bisulfite conversion in future single-cell DNA methylation profiling methods.

Another issue is that a clear standard analysis process has not been established. The gold standard for NGS processing of bisulfite sequencing data was presented as a constant pipeline through Bismark–Trim Galore!–Samtools, but for the interpretation of results after methylation of the individual CpG positions of each read is confirmed, numerous methods are still used. The root cause of this situation is the ambiguity of the meaning of each CpG methylation. DNA methylation is regulated by various biological factors such as TET, histones, MBD, transcription factors, and adjacent CpG status, and these factors change over a short or long period of time due to macroscopic factors such as excessive exercise and disease. The boundary line for the observed continuous variable, methylation level, is ambiguous regarding actual transcription control. This requires the analyst to spend extra time to validate the analyzed result. The accumulation of DNA methylation data is insufficient to consider all the variables described before. However, many of the existing methylome data are only a one-off event, and there are many cases in which each paper lacks information other than the features of interest to the researcher, thus limiting further use. In addition, the assignment of cell types in cluster visualization analysis of single-cell data, such as t-SNE, is difficult compared to single-cell RNA-seq data due to the lack of clear cell-type methylation markers compared to gene expression markers. To overcome these limitations, high-quality, reproducible data with minimized bias in each feature should be accumulated.

Because of these challenges, the current best method is to introduce a multi-omics method to cross-validate. It has been demonstrated in several experiments that this can be an effective alternative in the current situation where the understanding of methylation is limited due to a lack of data. In addition to the transcriptome and chromatin accessibility, cross-validation between additional elements and the DNA methylome will be a milestone for researchers leading to accurate interpretations of the data.

Nevertheless, we believe that much will be possible in the future using DNA methylation alone. One piece of good news is that the cost of data acquisition is gradually decreasing due to the development of NGS. Because the cost of sequencing is a bottleneck in whole-genome bisulfite sequencing studies, a reduction in sequencing cost would generate more whole-methylome data. A large amount of DNA methylation-related data held in various databases was created using several commercially available methylation capture panels, and this is the result of efforts to obtain the most meaningful data at a limited cost and accordingly a fixed amount of data. There are efforts to build marker sets between different cell types including DNA methylation of mouse brain [112] and discovery of DMRs for some tissue types [113]. In addition, the global consortium for studying the human epigenome, called the International Human Epigenome Consortium (IHEC), spends many resources to understand disease-related methylation patterns and the heterogeneity between different cell types. The accumulation of comprehensive data could provide an opportunity to understand methylation. The accumulation of evidence on methylation will make it possible to find methylation hotspot regions that fluctuate by different tissue types, different experimental or environmental conditions, and heterogeneous diseases such as cancer. Furthermore, the discovery of cell-type-specific markers through accumulated data would benefit the cellular heterogeneity analysis through visualization of single-cell DNA methylation data, including the assignment of cell clusters in a t-SNE plot. We believe that an understanding of the relationship between methylation and its biological role in disease will be revealed with further data in the future.

## Figures and Tables

**Table 1:** Brief summary of single-cell DNA methylation profiling methods ^1^.

Basis	PBAT	Single-Cell Method	Related Method ^2^	Advanced Strategy ^3^	Ref.
RRBS	No	scRRBS	None	Single-tube reaction, tRNA carrier	[46]
		Q-RRBS	scRRBS	UMI adaptor	[47]
		MID-RRBS	None	Microfluidics	[41]
		scTrio-seq	scRRBS, scRNA-seq	Multi-omics (RNA, CNV)	[48]
		scMT-seq	scRRBS, Smart-seq2	Multi-omics (RNA)	[49]
WGBS	Yes ^4^	scBS-seq	None	Single-tube reaction, Preamplification, SPRI bead	[50]
		scPBAT	None	Non-preamplification (repeat-specialized)	[51]
		scWGBS	None	Non-preamplification	[52]
		snmC-seq2 ^5^	snmC-seq	Single-strand library preparation method	[53]
		sci-MET	None	Transposase tagmentation, combinatorial indexing	[54]
		scTrio-seq2	scBS-seq, scTrio-seq	Multi-omics (RNA, CNV)	[55]
		scM&T-seq	scBS-seq, G&T-seq	Multi-omics (RNA)	[56]
		scNMT-seq	scM&T-seq, NOMe-seq	Multi-omics (RNA, chromatin accessibility)	[57]
		iscCOOL-seq ^5^	scCOOL-seq, NOMe-seq	Multi-omics (chromatin accessibility, CNV, ploidy)	[58]
		scNOMe-seq	NOMe-seq	Multi-omics (chromatin accessibility)	[59]
MSRE	No	scCGI-seq	None	MDA	[60]

^1^ This table originated from another review paper (Karemaker and Vermeulen, 2018) [61] and has been reduced, reorganized, and updated to fit our review scope. The original review contains descriptions of various methods that are not within the scope of this paper. ^2^ If there is no mention of a related method in the paper, even if the method is similar to other methods, the classification is None. ^3^ Because DNA methylation is indicated by default, it is not separately indicated in multi-omics. ^4^ If there was an adaptor tagging step after bisulfite conversion, it was classified as PBAT. ^5^ Because the improved method and the original method are similar in basic purpose, only the most recent method is indicated. PBAT: post-bisulfite adaptor tagging; RRBS: reduced representation bisulfite sequencing; Q: quantitative; sc: single-cell; UMI: unique molecular identifier; MID: microfluidic diffusion; trio, triple omics; WGBS: whole-genome bisulfite sequencing; CNV: copy number variation; MT: methylome and transcriptome; SPRI: solid-phase reversible immobilization; M&T: methylation and transcriptome; G&T: genome and transcriptome; NMT: nucleosome, methylation, and transcription; NOMe: nucleosome occupancy and methylome; iscCOOL-seq: improved single-cell chromatin overall omic-scale landscape sequencing; sci-MET: single-cell indexing for methylation analysis; MSRE: methylation-sensitive restriction enzymes; CGI: CpG island; MDA: multiple displacement amplification.

**Table 2:** Bisulfite sequencing read-alignment software programs ^1^.

Aligner	Index Method	URL
BSMAP	Wild-card	https://code.google.com/archive/p/bsmap/
RMAPBS	Wild-card	https://github.com/smithlabcode/rmap
Bismark	Three-letter	https://github.com/FelixKrueger/Bismark
BS-Seeker	Three-letter	version1: https://bmcbioinformatics.biomedcentral.com/articles/10.1186/1471-2105-11-203 version2: https://github.com/BSSeeker/BSseeker2 version3: https://github.com/khuang28jhu/bs3/
BitmapperBS	Three-letter	https://github.com/chhylp123/BitMapperBS

^1^ This table is based on the table of another review paper (Bock C., 2012) [82] and has been modified to include the well-known programs that fit the scope of this paper.
